# Clinical and intraoperative findings for dangerous chronic suppurative otitis media in paediatric cases

**DOI:** 10.11604/pamj.2021.40.174.22291

**Published:** 2021-11-21

**Authors:** Ratna Dwi Restuti, Harim Priyono, Rangga Rayendra Saleh, Ayu Astria Sriyana

**Affiliations:** 1 Otology Division, Ear Nose and Throat Department, Faculty of Medicine, Universitas Indonesia-Cipto Mangunkusumo General Hospital, Jakarta, Indonesia

**Keywords:** Pediatric dangerous CSOM, intraoperative findings, complication

## Abstract

The number of complications in dangerous Chronic Suppurative Otitis Media (CSOM) remain high, especially in late presentation. Extensive disease with intratemporal and intracranial complications, especially in children, was commonly found in Cipto Mangunkusumo General Hospital (CMGH), a tertiary referral hospital. This was a retrospective study that used secondary data from 2017 to 2019 in CMGH. All paediatric patients with diagnoses of dangerous CSOM and who underwent surgery from January 2017 to June 2019 were included. Clinical and intraoperative findings were described in this study. Twenty paediatric patients underwent surgery in CMGH from 2017 to 2019; of that number, 17 had unilateral dangerous CSOM, and three had bilateral dangerous CSOM. All patients aged 2 to 18 years old underwent canal wall down technique surgery. Profound hearing loss was found in nine ears; severe loss, in four ears; moderately severe loss, in four ears; and moderate loss, in seven ears. The most common intraoperative finding was total ossicular destruction in 17/23 ears. Erosion of sigmoid sinus plate was found in 5/23 ears, with perisinus abscess occurring in one case. Tegmen erosion was found in 4/23 ears. Facial nerve dehiscence was found in seven ears (5 vertical segments, 2 horizontal segments) and two patients had facial nerve paralysis before surgery. Lateral semicircular canal (SCC) fistula was found in 6/23 ears. Late presentation in dangerous CSOM can lead to extensive disease and complication, especially in paediatric patients.

## Introduction

Chronic suppurative otitis media (CSOM) is one of the most common childhood diseases worldwide. World Health Organization (WHO) estimates CSOM affected between 65 and 330 million people worldwide, mainly in developing countries. Long standing disease may result in chronic tympanic membrane perforation and cholesteatoma formation [1-3]. Extensive disease with severe complications, especially in children, was commonly found in Cipto Mangunkusumo General Hospital (CMGH), a tertiary referral hospital. Therefore, our study aims to evaluate clinical and intraoperative findings in pediatric cases.

## Methods

This study was a retrospective study using secondary data from 2017 to 2019 in CMGH. All pediatric patients with age range from 2 - 18 years and underwent surgery for dangerous type chronic suppurative otitis media in January 2017 to June 2019 were included. This period of time was chosen due to medical report completion and video recording availability. Data from medical records, including clinical data and operation reports were collected. In case of missing data of intraoperative finding in operation reports, we collected data from video recording. The main interest in intraoperative findings were the disease extension related to complication as well as common involved anatomical structure in pediatric dangerous type CSOM. The data collection was summarized in tables. Clinical and intraoperative findings were described in this study.

**Ethics approval:** this study has passed the Faculty of Medicine University of Indonesia Ethics Committee approval.

**Privacy and confidentiality of patient information:** the identifiable information and confidentiality of all patients were protected.

## Results

All patients aged from 2 to 18 years and diagnosed with dangerous CSOM were included in this study. Twenty pediatric patients underwent surgery in CMGH from 2017-2019; of that number, 17 had unilateral disease, and three had bilateral disease. A total of 23 ears diagnosed with dangerous CSOM were included in this study.

**Patient´s demographic and clinical data:** there were 20 pediatric patients with dangerous CSOM from 2017 to 2019. The male-to-female ratio was 1: 1.2. Patients were aged 2 to 18 years, with a mean age of 12.4 ± 4.2 years. Most patients had the illness for long duration: 1 to 5 years (50%) and more than 5 years (35%). Our study also found retroauricular abscess as the most common intratemporal complication (Table 1).

**Table 1:** demographic and clinical data of patients

Demographic data	Patients
**Gender**	
Male	9
Female	11
**Age**	
2-8 years old	3
8-18 years old	17
**Duration of illness**	
<1 year	3
1-5 years	10
>5 years	7
**Complication**	
Facial nerve paralysis	2
Cerebral abscess	1
Retroauricular abscess	6

**Preoperative hearing evaluation:** hearing impairment in our study ranged between moderate and profound hearing loss. Profound hearing loss was the most common finding (Table 2). Hearing impairment was the most common morbidity in CSOM patients. Our results revealed that more than half of our cases (12/23 ears) suffered severe to profound hearing loss (Table 2).

**Table 2:** hearing impairment severity

Degree of hearing impairment	Ears
Mild	0
moderate	7
Moderate-severe	4
Severe	3
Profound	9

**Intraoperative findings:** the extent of the disease was defined by the number of sites involved with cholesteatoma. According to Saleh and Mills´ classification based on intraoperative involvement of the attic, antrum, mastoid, mesotympanum, eustachian tube, labyrinth, and middle cranial fossa, cholesteatoma was classified as stage I (one site), stage II (two sites), stage III (three sites), stage IV (four sites), and stage V (five or more sites) [4]. Our study mostly discovered stage III cholesteatoma in 10 ears (43.4%) and stage V in six ears (26%). In our study, the most common sites of involvement was stage III (including epitympanum, antrum, and mesotympanum) as seen in Table 3. In stage V, despite involvement in three common sites, it was also involved in the mastoid, labyrinth, middle cranial fossa, and Eustachian tube.

**Table 3:** intraoperative findings of 20 pediatric patients (age 2-18 years) who underwent chronic suppurative otitis media surgery in Cipto Mangunkusumo General Hospital from 2017 - 2019

Intraoperative findings	Ears
**Semi-circular canal fistula**	
Lateral semi-circular canal fistula	6
No erosion	17
**Tegmen erosion**	
Erosion	4
No erosion	19
**Sigmoid sinus plate**	
Erosion	5
No erosion	18
Perisinus abscess	1
**Facial nerve dehiscense**	
Pars mastoid	5
Pars tympani	2
**Ossicular chain findings**	
Absent ossicular chain	17
Erosion (in one or more ossicles)	2
Intact ossicular chain	4
**Extent of disease**	
Stage I	0
Stage II	1
Stage III	10
Stage IV	3
Stage V	6

In 23 ears, the most common intraoperative findings were total ossicular destruction. Total ossicular chain destruction was found in 17 ears (73%), whereas only four ears had intact ossicular chains (Table 3). Erosion in one or more ossicles were found in two ears. The incus and head of malleus were eroded in these two patients. This study also found semicircular canal (SCC) fistula in six lateral SCCs (26%), and five from those ears with SCC fistula (83%) displayed profound hearing loss, regardless of the type of hearing loss (Table 3).

Tegmen erosion was found in four ears (17.3%), and one of these patients had intracranial complication of temporal brain abscess which was confirmed with brain computed tomography (CT) scan with contrast (Table 3). Sigmoid sinus plate erosion was found in five ears (21.7%). There was granulation tissue over the sigmoid sinus and perisinus abscess in one ear (4%). Facial nerve was exposed in 30.4% ears, and the most affected site was the mastoid segment (Table 3). Facial palsy was found in two out of five cases with exposed mastoid segment facial nerve.

## Discussion

Hearing impairment is the most common morbidity associated with CSOM [5]. Our study found that most patients with long-standing disease had profound hearing loss, regardless of the type of hearing loss. Infection of the middle ear leads to the formation of inflammatory mediators that can morphologically and functionally change the auditory structure. These inflammatory mediators can penetrate the round window membrane and cause cochlear damage. Other studies have also demonstrated that bacterial toxins found in the middle ear during CSOM can pass into the cochlea and result in cochlear damage. These bacterial toxins can be exotoxins produced by both gram-positive and gram-negative bacteria or endotoxins. These toxins might cause damage to hair cells. A significant loss of outer and inner hair cells and significant atrophy of the striae vascularis in the basal turn of the cochlea has been observed in CSOM patients [6,7]. Unsurprisingly, long-standing illness led to extensive disease and morbidity.

Almost all of our patients with SCC fistula had profound hearing loss (5/6 ears). As the SCC is connected to the cochlea, a labyrinthine fistula can lead to sensorineural hearing loss. Preoperative deafness according to several studies ranges from approximately 12% to 30% [8]. The higher rate in our series may be caused by longer duration of CSOM compared to other studies. The most common SCC involvement in our study was lateral SSC. This was similar with another study in which the most common affected SCC by fistula was lateral SCC, followed by promontorium, superior SCC, and posterior SCC [9]. One of four ears (25%) with tegmen erosion in our study had intracranial complications due to extensive disease. Tegmen erosion with dura exposure increases the risk of intracranial complication due to the contiguous spreading of disease. As stated in the literature, otogenic brain abscesses are commonly located in the temporal lobe and cerebellum, which is explained by the contiguous spread of the disease process [10].

Children with cholesteatoma demonstrate more aggressive disease than adults with higher recurrence rates [11]. The ossicular chain was found absent in most ears (73%) in our study. This finding suggested the aggressive nature of the disease in pediatric cases. Absent ossicular chain (total destruction) was found in three patients with disease duration less than 6 months. Footplate was left intact in most cases but mostly covered with granulation tissue. The most intact ossicle was stapes, and it was the last to be eroded. The present study uncovered multiossicular damage of ossicles, which is similar to the results of previously reported study by Shirazi [12]. Ossicular chain involvement at the time of presentation was associated with more extensive disease as seen by Shirazi and colleagues. They also found that ossicular chain involvement was a significant predictor of high risk for recurrent disease; therefore, more aggressive surgical disease eradication procedures are needed in these patients [12]. Disease eradication using canal wall down technique was the most frequent treatment in our pediatric cases, considering the high risk of recurrence and aggressive features. Facial nerve paralysis was found in two patients and intraoperatively the mastoid segment facial nerves were exposed and covered with granulation. The mastoid segment was the most common affected site in our study. These findings appear to differ with other studies conducted by Choi *et al*. who found the tympanic segment to be the most frequently affected site and who also revealed that patients with facial paralysis displayed more frequent destruction of the bony labyrinth and cranial base. Both of our patients with facial nerve paralysis had an intact SCC and cranial base. Facial canal dehiscence was observed in 30.4% ears in our study.

This finding differed with another study that found 52% of middle ear cholesteatoma patients had facial canal dehiscence. In the literature, facial paralysis incidence reportedly ranged from 1% to 3% in patients with chronic otitis media [13,14]. The confirmed aetiology of facial nerve paralysis in CSOM is not well known. Direct inflammatory involvement of the facial nerve through fallopian canal dehiscence and compression resulting from edema are believed to be the pathophysiology of this effect. The cholesteatoma itself might cause facial paralysis through neurotoxic substances or bony destruction through various enzymatic activities [15]. Furthermore, a histopathological study revealed degenerative and inflammatory changes of the facial nerve in patients with facial paralysis due to CSOM [16]. However, early diagnosis and treatment of cholesteatoma remain the mainstay of treatment. The prognosis of facial paralysis is related to the time of intervention. Both of our patients had facial nerve paralysis more than 2 months after onset with House Brackmann grade 4 at the time of presentation, and the patients did not fully recover after surgery. Our findings were consistent with another study that found surgery more than 2 months after the onset of paralysis resulted in poorer outcomes [17].

Our patients were presented mostly in the late stage, and our intraoperative findings confirmed extension and destruction in mastoid and tympanic cavity area. All of the patients in stage V had the illness for a long time, with a minimum of 3 years and maximum of 17 years duration. Three cases with less than 6 months of persistent ottorhea duration also had stages III and IV. Cholesteatoma and granulation findings were found intraoperatively in the epitympanum, mesotympanum, antrum, and mastoid with ossicular destruction. These findings demonstrated the aggressiveness of cholesteatoma features in pediatric populations. Similar to other studies, we uncovered more aggressive disease in younger children who had shorter disease duration and were also found upon examination to have active persistent ear discharge. Intraoperatively, AlQudehy *et al*. also found granulation tissues in the sinus tympani, epitympanum, and medial to the ossicles, along with ossicular erosion, and they were predictors of aggressive disease [11]. The limitations of our study are that we did not evaluate pre-operative radiological features (high resolution computed tomography mastoid) as additional data and this should be considered for future research.

## Conclusion

Late presentation in dangerous CSOM may lead to extensive disease especially in pediatric patients. Hearing impairment in patients and complications were clinically related with intraoperative findings. Early diagnosis and treatment are mandatory to prevent extensive disease and complications.

## References

[1] Li MG, Hotez PJ, Vrabec JT, Donovan DT (2015). Is chronic suppurative otitis media a neglected tropical disease. PLoS Negl Trop Dis.

[2] Orji FT (2013). A survey of the burden of management of chronic suppurative otitis media in a developing country. Ann Med Health Sci Res.

[3] Acuin J (2004). Chronic suppurative otitis media: burden of illness and management options.

[4] Saleh HA, Mills RP (1999). Classification and staging of cholesteatoma. Clin Otolaryngol Allied Sci.

[5] Aarhus L, Tambs K, Kvestad E, Engdahl B (2015). Childhood otitis media: a cohort study with 30-year follow-up of hearing (The HUNT Study). Ear Hear.

[6] Kolo ES, Salisu AD, Yaro AM, Nwaorgu OG (2012). Sensorineural hearing loss in patients with chronic suppurative otitis media. Indian J Otolaryngol Head Neck Surg.

[7] Joglekar S, Morita N, Cureoglu S, Schachern PA, Deroee AF, Tsuprun V (2010). Cochlear pathology in human temporal bones with otitis media. Acta Otolaryngol.

[8] Geerse S, Wolf MJF, Ebbens FA, Spronsen E (2017). Management of labyrinthine fistula: hearing preservation versus prevention of residual disease. Eur Arch Otorhinolaryngol.

[9] Copeland BJ, Buchman CA (2003). Management of labyrinthine fistulae in chronic ear surgery. Am J Otolaryngol.

[10] Ryan JT, Pena M, Zalzal GH, Preciado DA (2016). Otogenic lateral sinus thrombosis in children: a review of 7 cases. Ear Nose Throat J.

[11] Alqudehy ZA, Al-Abdulqader A, Alsyaikh H (2016). Pediatric cholesteatoma aggressiveness; a comparative study of predictive factors and recurrence rate. Otolaryngol Open Access J.

[12] Shirazi MA, Muzaffar K, Leonetti JP, Marzo S (2006). Surgical treatment of paediatric cholesteatomas. Laryngoscope.

[13] Yetiser S, Tosun F, Kazkayasi M (2002). Facial nerve paralysis due to chronic otitis media. Otol Neurotol.

[14] Choi JW, Park YH (2015). Facial nerve paralysis in patients with chronic ear infections: surgical outcomes and radiologic analysis. Clin Exp Otorhinolaryngol.

[15] Chu FW, Jackler RK (1988). Anterior epitympanic cholesteatoma with facial paralysis: a characteristic growth pattern. Laryngoscope.

[16] Fuse T, Tada Y, Aoyagi M, Sugai Y (1996). CT detection of facial canal dehiscence and semicircular canal fistula: comparison with surgical findings. J Comput Assist Tomogr.

[17] Ikeda M, Nakazato H, Onoda K, Hirai R, Kida A (2006). Facial nerve paralysis caused by middle ear cholesteatoma and effects of surgical intervention. Acta Otolaryngol.

